# Association between prehospital adrenaline administration and short-term outcomes in patients with shockable out-of-hospital cardiac arrest undergoing extracorporeal cardiopulmonary resuscitation: a propensity-score matched analysis

**DOI:** 10.1016/j.ijcha.2025.101735

**Published:** 2025-06-30

**Authors:** Shoji Kawakami, Hidenobu Koga, Tetsuhisa Yamada, Jun-Ichiro Nishi

**Affiliations:** a Department of Cardiology, Aso Iizuka Hospital, Fukuoka, Japan; b Clinical Research Support Office, Aso Iizuka Hospital, Fukuoka, Japan; c Department of Emergency Medicine, Aso Iizuka Hospital, Fukuoka, Japan

**Keywords:** Cardiac arrest, Extracorporeal resuscitation, Adrenaline, Epinephrine

## Abstract

**Background:**

In patients with out-of-hospital cardiac arrest (OHCA) and an initial shockable rhythm undergoing extracorporeal resuscitation (ECPR), the effect of adrenaline on neurological outcomes remains uncertain. This study aimed to investigate the association between prehospital adrenaline and clinical outcomes in this patient population.

**Methods:**

This multicentre, prospective study (JAAM-OHCA registry) enrolled 81,234 patients with OHCA between 2014 and 2021. Patients with an initial shockable rhythm who underwent ECPR for a cardiac cause were eligible for this study. The primary outcome was a favourable neurological outcome at 30 days. The secondary outcome was prehospital return of spontaneous circulation (ROSC) or 30-day survival. A propensity score-matched analysis was performed to adjust for confounding and evaluate the independent association between prehospital adrenaline and study outcomes.

**Results:**

Among 1,061 patients, 442 (41.7 %) received prehospital adrenaline and 619 (58.3 %) did not. In 329 matched pairs, the prehospital ROSC rate was significantly higher in the adrenaline group (30 [9 %] vs 16 [5 %]; adjusted odds ratio [OR] 1.96, 95 % confidence interval [CI] 1.05–3.67, *P* = 0.03). However, 30-day survival (70 [21 %] vs 77 [23 %]; adjusted OR 0.88, 95 % CI 0.61–1.28, *P* = 0.51) and a favourable neurological outcome at 30 days (24 [7 %] vs 30 [9 %]; adjusted OR 0.78, 95 % CI 0.45–1.37, *P* = 0.39) were not significantly different.

**Conclusions:**

In patients with OHCA and an initial shockable rhythm who underwent ECPR, prehospital adrenaline was significantly associated with increased prehospital ROSC, but not with increased in survival and a favourable neurological outcome at 30 days.

## Graphical abstract

**Figure:**
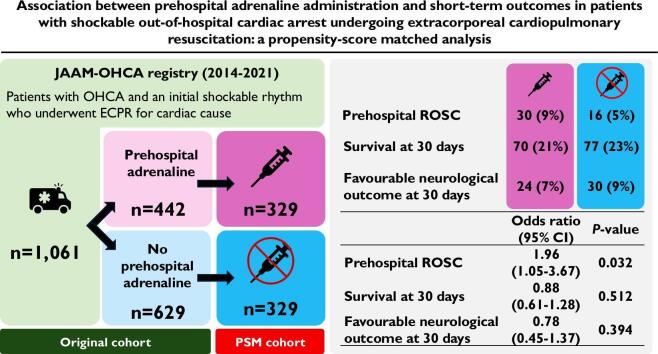

## 1 Introduction

Current guidelines recommend prompt defibrillation for patients with shockable rhythms, such as ventricular fibrillation or pulseless ventricular tachycardia. Adrenaline is typically reserved for patients with persistent shockable rhythms [1,2]. Although adrenaline administration has been shown to increase the likelihood of return of spontaneous circulation (ROSC), its impact on survival with favourable neurological outcomes remains uncertain [[3], [4], [5]]. Extracorporeal cardiopulmonary resuscitation (ECPR) using veno-arterial extracorporeal membrane oxygenation (V-A ECMO) provides effective circulatory and respiratory support in patients with out-of-hospital cardiac arrest (OHCA) resulting from reversible causes, potentially improving survival and neurological outcomes [[6], [7], [8]]. However, in patients undergoing ECPR, the absolute benefit of adrenaline in achieving favourable neurological outcomes is expected to be smaller. Despite this, the precise relationship between prehospital adrenaline administration and clinical outcomes in such patients remains unclear. To address this knowledge gap, we utilized the Japanese Association for Acute Medicine (JAAM)-OHCA Registry, a multicentre, prospective registry that enrols patients with OHCA who are transported to critical care medical centres or hospitals with emergency care departments in Japan [9]. This study aimed to examine the association between prehospital adrenaline administration and clinical outcomes in patients with OHCA who had an initial shockable rhythm and underwent ECPR.

## 2 Methods

### 2.1 Data collection and study design

This study involved a retrospective analysis of the JAAM-OHCA registry, a prospective, multicentre, nationwide database established by the steering committee of the JAAM. Details of the registry have been previously published [8,9]. Between June 2014 and December 2021, the JAAM-OHCA registry enrolled patients with OHCA for whom resuscitation was attempted and who were transported to 102 participating institutions. In-hospital data from the JAAM-OHCA registry were systemically merged with prehospital data from the All-Japan Utstein Registry of the Fire and Disaster Management Agency (FDMA) of Japan.

**Fig. 1:**
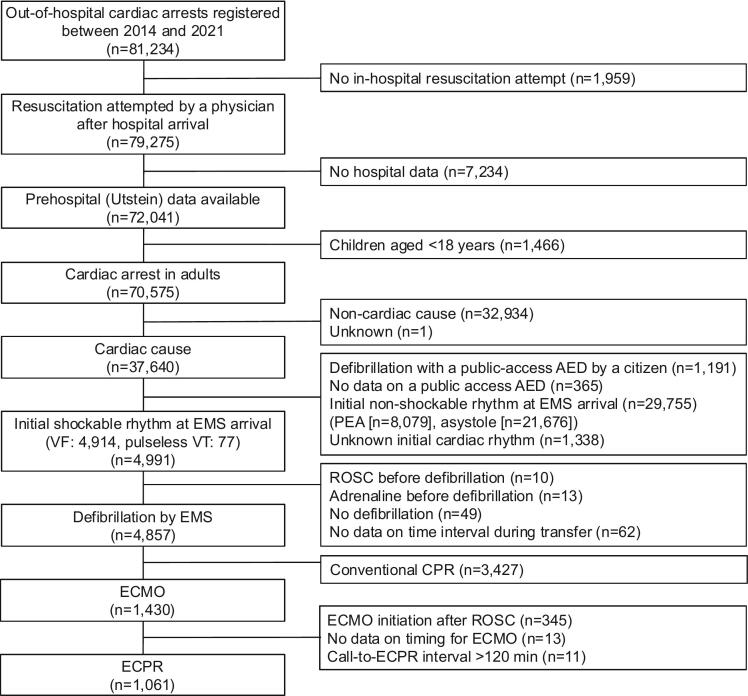
Study flow. AED, automated external defibrillator; PEA, pulseless electrical activity; EMS, emergency medical services; VF, ventricular fibrillation; VT, ventricular tachycardia; ROSC, return of spontaneous circulation; CPR, cardiopulmonary resuscitation; ECMO, extracorporeal membrane oxygenation; ECPR, extracorporeal cardiopulmonary resuscitation.

The study included adult patients with OHCA of cardiac origin who had an initial shockable rhythm at the time of emergency medical services (EMS) arrival, underwent defibrillation at the scene, and received ECPR after hospital arrival (Fig. 1). Patients were excluded if any of the following criteria were met: age under 18 years, resuscitation not attempted, a citizen performed defibrillation using a public access automated external defibrillator, an initial non-shockable rhythm on EMS arrival, defibrillation not performed, ROSC achieved or adrenaline administration before first defibrillation, ECPR not performed.

Prehospital data based on Utstein-style international guidelines for reporting OHCA were recorded. In brief, sex was recorded as biological male or female. Age, aetiology of arrest, bystander status, presence or absence of bystander CPR, presence or absence of dispatcher-assisted CPR, initial electrocardiogram rhythm, and treatments performed by EMS personnel were also recorded. Prehospital ROSC was defined as any spontaneous, palpable pulse confirmed by cardiac rhythm monitoring prior to hospital arrival [10]. ECPR was defined as the initiation of V-A ECMO during ongoing CPR, regardless of whether transient ROSC occurred between EMS contact and V-A ECMO initiation. Patients who achieved sustained ROSC and subsequently received V-A ECMO without ongoing CPR were excluded. All included patients were undergoing CPR at the time of V-A ECMO initiation.

The primary outcome was a favourable neurological outcome, defined as a Cerebral Performance Category (CPC) score of 1 or 2 at 30 days. The secondary outcome was prehospital ROSC or survival at 30 days. The CPC was evaluated by clinicians or research assistants in each hospital and registered in the database.

The study protocol and retrospective analysis were approved by the Ethics Committee of Kyoto University (R1045), the Aso Iizuka Hospital (22091), and each participating institution. The requirement for written informed consent was waived for this observational study.

### 2.2 Field protocol for EMS in Japan

Details of the Japanese emergency system have been discussed previously [[10], [11], [12], [13]]. In brief, the FDMA manages a single emergency network, with ambulance services that cover the entire country of Japan. All EMS personnel are trained to perform CPR according to Japan Resuscitation Council guidelines, which are based on the International Liaison Committee on Resuscitation guidelines. In general, an ambulance crew consists of three EMS personnel, one of whom is an emergency lifesaving technician. With direct online medical direction, they administer adrenaline to patients aged > 8 years with pulseless electrical activity, ventricular fibrillation, or pulseless ventricular tachycardia rhythms after defibrillation or to those with witnessed asystole.

### 2.3 Data analysis

Patients were divided into two groups: those who were administered prehospital adrenaline by EMS and those were not. We then compared the baseline characteristics, prehospital and in-hospital data, and clinical outcomes in the unadjusted and propensity score-matched cohorts. The call-to-adrenaline interval was defined as the interval from emergency call receipt to the first prehospital adrenaline administration by EMS. The call-to-ECPR interval was defined as the interval from emergency call receipt to the initiation of circulatory support by VA-ECMO.

### 2.4 Statical analysis

Statistical analyses were conducted using JMP17.2.0 (SAS Institute Japan, Tokyo, Japan). Restricted cubic spline curves were created using Python 3.10.12 (Python Software Foundation, Wilmington, DE, US). Statistical significance was set at *P* < 0.05. Data are expressed as median (interquartile range [IQR]). Intergroup comparisons of continuous variables were performed using the Wilcoxon rank sum test. Nominal variables were compared using the χ^2^ test or Fisher’s exact test. The propensity score was estimated using a logistic regression model that adjusted for age, sex, witnessed arrest, bystander-initiated CPR, dispatcher-assisted CPR, advanced airway management by EMS, EMS call-to-defibrillation interval, year, and district of Japan. Propensity score matching (PSM) was performed for patients who received prehospital adrenaline from EMS and those who did not receive, at a 1:1 ratio, using the nearest-neighbour matching method within a calliper of 0.20 of the propensity score. We used the standardised difference to measure covariate balance, whereby an absolute standardised difference above 10 % represented meaningful imbalance.

## 3 Results

### 3.1 Study participants

During the study period, 81,234 patients were registered. After applying the exclusion criteria, 1,061 patients who had an initial shockable rhythm at EMS arrival and who underwent ECPR were eligible (Fig. 1). Of these, 112 (10.6 %) patients had a favourable neurological outcome at 30 days and 949 (89.4 %) did not. The distribution of patients by CPC was as follows: CPC 1, 73 (6.9 %) patients; CPC 2, 39 (3.7 %) patients; CPC 3, 50 (4.7 %) patients; CPC 4, 95 (9.0 %) patients; and CPC 5, 804 (75.8 %) patients.

### 3.2 Clinical characteristics and prognosis by prehospital adrenaline administration

**Fig. 2:**
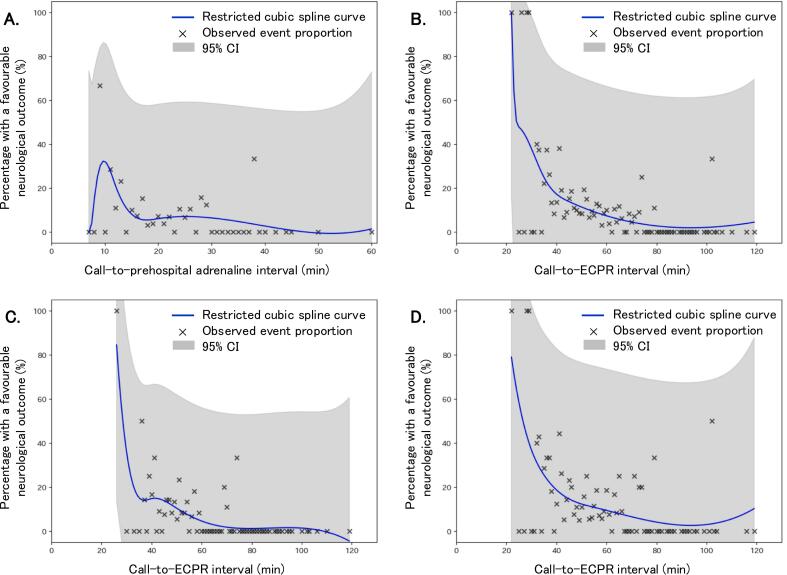
Restricted cubic spline curves for the association between intervals and a favourable neurological outcome at 30 days. (A) call-to-prehospital adrenaline interval, (B) call-to-ECPR interval in the overall cohort, (C) call-to-ECPR interval in the prehospital adrenaline group, (D) call-to-ECPR interval in the group without prehospital adrenaline. ECPR, extracorporeal cardiopulmonary resuscitation, CI, confidence interval.

Of 1,061 patients, 442 (41.7 %) received prehospital adrenaline and 619 (58.3 %) did not. Fig. 2 presents restricted cubic spline curves for the association between the call-to-prehospital adrenaline interval (Fig. 2A) or the call-to-ECPR interval (Fig. 2B) and a favourable neurological outcome at 30 days. Regardless of whether the patient received adrenaline (Fig. 2C) or not (Fig. 2D), the proportion of patients with a favourable neurological outcome at 30 days decreased as the call-to-ECPR interval prolonged. Table 1 presents the baseline characteristics of eligible patients, stratified by prehospital adrenaline administration. PSM on the nine variables matched 329 (74.4 %) of 442 patients who received prehospital adrenaline with 329 (53.2 %) of 619 patients who did not receive prehospital adrenaline. Matching successfully achieved covariate balance, as shown by a standardized difference of < 10 % for all variables.

**Table 1:** Baseline characteristics of patients who received or did not receive prehospital adrenaline.

		**Unadjusted**			Propensity score-matched population*			
	**Total (n = 1,061)**	**Prehospital adrenaline (n = 442)**	**No prehospital adrenaline (n = 619)**	***P*-value**	**Prehospital adrenaline (n = 329)**	**No prehospital adrenaline (n = 329)**	***P*-value**	**SMD**
Age, years	59 (48–69)	59 (49–69)	59 (48–68)	0.207	59 (49–70)	59 (48–68)	0.746	0.016
Male	926 (87)	392 (89)	534 (86)	0.244	289 (88)	290 (88)	0.905	0.010
Witnessed arrest	828 (78)	347 (79)	481 (78)	0.756	258 (78)	257 (78)	0.925	0.007
Bystander-initiated CPR	558 (53)	241 (55)	317 (51)	0.287	177 (54)	172 (52)	0.696	0.030
Dispatcher-assisted CPR	541 (52)	245 (56)	296 (48)	0.016	173 (53)	164 (50)	0.483	0.055
Advanced airway management	616 (68)	322 (80)	294 (59)	<0.001	252 (77)	250 (76)	0.855	0.014
Call-to-defibrillation by EMS personnel interval, minutes	10 (8–12)	10 (9–12)	10 (8–12)	0.860	10 (9–12)	10 (8–12)	0.391	0.023

Year				0.034			0.997	
2014	52 (5)	18 (4)	34 (5)	0.291	15 (5)	17 (5)	0.717	0.028
2015	129 (12)	49 (11)	80 (13)	0.366	42 (13)	43 (13)	0.908	0.009
2016	150 (14)	62 (14)	88 (14)	0.931	47 (14)	47 (14)	1.000	0.000
2017	138 (13)	65 (15)	73 (12)	0.164	42 (13)	41 (12)	0.907	0.009
2018	171 (16)	75 (17)	96 (16)	0.524	53 (16)	55 (17)	0.833	0.016
2019	139 (13)	63 (14)	76 (12)	0.347	42 (13)	42 (13)	1.000	0.000
2020	162 (15)	76 (17)	86 (14)	0.141	58 (18)	60 (18)	0.839	0.016
2021	120 (11)	34 (8)	86 (14)	0.002	30 (9)	24 (7)	0.394	0.066

District				<0.001			0.951	
Hokkaido	139 (13)	70 (16)	69 (11)	0.026	60 (18)	58 (18)	0.839	0.016
Tohoku	80 (8)	24 (5)	56 (9)	0.028	21 (6)	18 (5)	0.620	0.039
Kanto	257 (24)	98 (22)	159 (26)	0.188	83 (25)	85 (26)	0.858	0.014
Chubu	112 (11)	68 (15)	44 (7)	<0.001	41 (12)	35 (11)	0.464	0.057
Kinki	413 (39)	162 (37)	251 (41)	0.199	111 (34)	120 (36)	0.462	0.057
Chugoku	25 (2)	3 (0.7)	22 (4)	0.002	1 (0.3)	0 (0)	0.317	0.078
Shikoku	2 (0.3)	1 (0.2)	2 (0.3)	1.000	1 (0.3)	1 (0.3)	1.000	0.000
Kyushu	32 (3)	16 (4)	16 (3)	0.331	11 (3)	12 (4)	0.832	0.017

Data are presented as median (interquartile range) or number (%). SMD, standardized mean difference; CPR, cardiopulmonary resuscitation; EMS, emergency medical service. *Adjusted for age, sex, witnessed arrest, bystander-initiated CPR, dispatcher-assisted CPR, advanced airway management, call-to-defibrillation by EMS personnel interval, year and district.

**Fig. 3:**
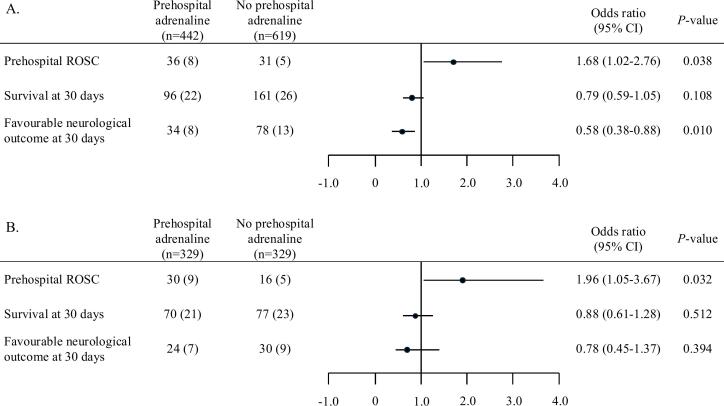
Effect of prehospital adrenaline on primary and secondary outcomes. Data are presented as number (%). CI, confidence interval; ROSC, return of spontaneous circulation.

Fig. 2 shows the proportion of prehospital ROSC, survival at 30 days, and a favourable neurological outcome at 30 days for patients who received prehospital adrenaline compared with those who did not. In the unadjusted cohort, the group that received prehospital adrenaline had a significantly lower proportion of prehospital ROSC (36 [8 %] vs 31 [5 %]; adjusted odds ratio [OR] 1.68 [95 % confidence interval, CI, 1.02–2.76], *P* = 0.04) and a favorable neurological outcome at 30 days (34 [8 %] vs 78 [13 %]; adjusted OR 0.58 [95 % CI 0.38–0.88], *P* = 0.01) than the group that did not, with no significant difference in survival at 30 days (96 [22 %] vs 161 [26 %]; adjusted OR 0.79 [95 % CI 0.59–1.05], *P* = 0.11, Fig. 3A). Consistent with the results from the unadjusted cohort, PSM showed that the group that received prehospital adrenaline had a significantly higher proportion of prehospital ROSC than the group that did not (30 [9 %] vs 16 [5 %]; adjusted OR 1.96 [95 % CI 1.05–3.67], *P* = 0.03). However, no significant differences were observed in the proportion of survival (70 [21 %] vs 77 [23 %]; adjusted OR 0.88 [95 % CI 0.61–1.28], *P* = 0.51) and a favorable neurological outcome at 30 days (24 [7 %] vs 30 [9 %]; adjusted OR 0.78 [95 % CI 0.45–1.37], *P* = 0.39, Fig. 3B). A post-hoc power analysis revealed that the statistical power to detect a difference in the primary outcome between the two groups was 13.4 %, based on the observed effect size and sample size.

Table 2 presents the time intervals and clinical characteristics during in-hospital resuscitation care for patients who received or did not receive prehospital adrenaline. Consistent with the results from the unadjusted cohort, PSM showed that patients who received prehospital adrenaline had a significantly longer defibrillation-to-hospital arrival interval (23 [18–30] vs 20 [15–26], *P* < 0.001), lower pH (6.90 [6.81–7.01] vs 6.95 [6.88–7.03], *P* < 0.001), and higher lactate levels at hospital arrival (120.6 [96.0–144.0] vs 110.0 [85.5–136.8], *P* = 0.007) than those who did not. However, no significant differences were observed in the hospital arrival-to-ECPR interval or the implementation of percutaneous coronary intervention and mechanical circulatory support.

**Table 2:** Time intervals and clinical characteristics of in-hospital resuscitation care in patients who received or did not receive prehospital adrenaline.

		**Unadjusted**			**Propensity score-matched**		
	**Total (n = 1,061)**	**Prehospital adrenaline (n = 442)**	**No prehospital adrenaline (n = 619)**	**P-value**	**Prehospital adrenaline (n = 329)**	**No prehospital adrenaline (n = 329)**	**P-value**
Defibrillation-to-hospital arrival interval, minutes	20 (15–27)	23 (18–29)	18 (13–24)	<0.001	23 (18–30)	20 (15–26)	<0.001
Hospital arrival-to-ECPR interval, minutes	26 (18–36)	25 (18–34)	26 (19–37)	0.068	26 (18–34)	26 (19–37)	0.267
Defibrillation after hospital arrival	827 (78)	340 (77)	487 (79)	0.497	247 (75)	250 (76)	0.786
Shockable rhythm at hospital arrival	684 (64)	307 (69)	377 (61)	0.004	221 (67)	200 (61)	0.088

Arterial blood gas at hospital arrival							
pH	6.94 (6.84–7.03)	6.91 (6.81–7.01)	7.0 (6.87–7.04)	<0.001	6.90 (6.81–7.01)	6.95 (6.88–7.03)	<0.001
Lactate, mg/dl	116.1 (90–144.0)	121.8 (96.1–145.4)	111.0 (84.6–136.8)	0.013	120.6 (96.0–144.0)	110.0 (85.5–136.8)	0.007

Drug administration after hospital arrival							
Adrenaline	946 (90)	385 (88)	561 (92)	0.010	296 (91)	295 (91)	0.815
Amiodarone	677 (65)	277 (63)	400 (66)	0.307	212 (65)	226 (71)	0.115
Coronary angiography	833 (79)	329 (74)	504 (81)	0.006	244 (74)	260 (79)	0.141
Percutaneous coronary intervention	482 (45)	183 (41)	299 (48)	0.026	140 (43)	161 (49)	0.100
Intra-aortic balloon pump implantation	666 (63)	268 (61)	398 (64)	0.224	205 (62)	218 (66)	0.290
Target temperature management	506 (48)	186 (42)	320 (52)	0.002	140 (43)	153 (47)	0.308

Data are presented as median (interquartile range) or number (%). ECPR, extracorporeal cardiopulmonary resuscitation. *Adjusted for age, sex, witnessed arrest, bystander-initiated CPR, dispatcher-assisted CPR, advanced airway management, call-to-defibrillation by EMS personnel interval, year and district.

## 4 Discussion

This study analyzed a multicenter, prospective registry, focusing on patients with OHCA and an initial shockable rhythm at EMS arrival who underwent ECPR, comparing those who received prehospital adrenaline with those who did not. The main findings from the propensity-score matching cohort were as follows: (i) the proportion of prehospital ROSC among patients who received prehospital adrenaline was significantly higher than that among those who did not; (ii) no significant differences were observed in the proportion of a favorable neurological outcome and survival at 30 days between the patients who received prehospital adrenaline and those who did not; (iii) patients who received prehospital adrenaline had a significantly longer defibrillation-to-hospital arrival interval, a significantly lower pH, and higher lactate levels than those who did not.

### 4.1 Effect of prehospital adrenaline during cardiopulmonary resuscitation in patients undergoing extracorporeal cardiopulmonary resuscitation

A systematic review and meta-analysis found that intravenous adrenaline improved survival to hospital admission and 3-month survival, but its effect on favourable neurological outcomes remained unclear among patients with OHCA and an initial shockable rhythm [[3], [4], [5]]. Adrenaline can impair heart function through β-adrenergic stimulation, causing dysrhythmias, increased oxygen demand, and a higher risk of cardiac arrest. α-adrenergic stimulation activates platelets, promoting thrombosis and impairing microvascular blood flow, worsening cerebral ischemia during CPR and after ROSC [3,[14], [15], [16]]. In addition to its pharmacological effects, the failure of adrenaline to improve neurological outcomes in patients with OHCA has also been associated with delays in transfer time caused by its administration, as reported in some studies [17,18].

Few studies have examined the effectiveness of adrenaline administration in patients undergoing ECPR. In a single-center, small-scale study, Shin et al. reported that lower total adrenaline doses during CPR in 30 patients with OHCA who underwent ECPR were associated with worse neurological outcomes at 6 months, although the cohort included both shockable and non-shockable rhythms [19]. In a secondary analysis of the SAVE-J II study, Yumoto et al. reported that prehospital adrenaline administration in 1289 patients with OHCA who underwent ECPR was associated with poorer neurological prognosis at hospital discharge, even among those with an initial shockable rhythm [17]. In our study, although prehospital adrenaline was associated with a higher rate of ROSC, it did not improve neurological outcomes. Patients who received prehospital adrenaline also had significantly longer defibrillation-to-hospital arrival intervals—likely due to extended on-scene interventions such as vascular access or drug delivery—and exhibited lower pH and higher lactate levels on hospital arrival, indicating more severe systemic hypoperfusion. Taken together, these findings suggest that while adrenaline may facilitate early ROSC, its potential benefits could be offset by adverse physiological effects and transport delays. Given that patients with OHCA and an initial shockable rhythm are considered good candidates for ECPR [4,[20], [21], [22]], our findings support continued adherence to current resuscitation guidelines [1,2,4] while emphasizing the importance of minimizing treatment-related delays. Timely initiation of ECPR is critical to improving outcomes [8,22], and it is desirable to administer prehospital adrenaline without delaying patient transfer.

### 4.2 Study limitations

This study has several potential limitations. First, as a retrospective analysis, unmeasured confounders may have influenced patient outcomes. Despite the use of PSM to adjust for baseline differences, residual confounding due to unaccounted variables cannot be ruled out. Second, because the study population was limited to adult patients with an initial shockable rhythm who underwent ECPR, individuals who achieved ROSC following adrenaline administration and thus did not require ECPR were excluded. This may have introduced selection bias, potentially enriching the study cohort with patients who were either unresponsive to adrenaline or in whom adrenaline was not administered. Accordingly, our findings should be interpreted within this selected ECPR population, which may limit their generalizability to broader OHCA populations, particularly those with non-shockable rhythms or who did not undergo ECPR. Third, the data were collected from a registry based on the Japanese emergency medical system. Therefore, the findings may not be directly applicable to regions with different medical protocols or infrastructure. Fourth, the statistical power to detect a meaningful difference in the primary outcome between patients who received prehospital adrenaline and those who did not was low (13.4 %). This low power may potentially limit the reliability of the findings regarding neurological outcomes at 30 days. Fifth, more than 90 % of both patients who received prehospital adrenaline and those who did not were administered adrenaline after hospital arrival, and this may have affected the results.

## 5 Conclusion

In patients with OHCA and an initial shockable rhythm who underwent ECPR, prehospital adrenaline was significantly associated with increased prehospital ROSC, but not with increased in survival and a favourable neurological outcome at 30 days.

## CRediT authorship contribution statement

**Shoji Kawakami:** Writing – review & editing, Writing – original draft, Visualization, Methodology, Investigation, Data curation, Conceptualization. **Hidenobu Koga:** Writing – review & editing, Formal analysis. **Tetsuhisa Yamada:** Supervision, Data curation. **Jun-Ichiro Nishi:** Supervision, Investigation.

## Declaration of competing interest

The authors declare that they have no known competing financial interests or personal relationships that could have appeared to influence the work reported in this paper.
